# Cost-Effectiveness Analysis of Administering Tranexamic Acid to Bleeding Trauma Patients Using Evidence from the CRASH-2 Trial

**DOI:** 10.1371/journal.pone.0018987

**Published:** 2011-05-03

**Authors:** Carla Guerriero, John Cairns, Pablo Perel, Haleema Shakur, Ian Roberts, on behalf of CRASH 2 trial collaborators

**Affiliations:** 1 Department of Health Services Research and Policy, London School of Hygiene and Tropical Medicine, London, United Kingdom; 2 Clinical Trials Unit, London School of Hygiene and Tropical Medicine, London, United Kingdom; University of Colorado Denver, United States of America

## Abstract

**Objective:**

To assess the cost effectiveness of giving tranexamic acid (TXA) to bleeding trauma patients in low, middle and high income settings.

**Methods:**

The CRASH-2 trial showed that TXA administration reduces the risk of death in bleeding trauma patients with a small but statistically significant increase in non-intensive care stay. A Markov model was used to assess the cost effectiveness of TXA in Tanzania, India and the United Kingdom (UK). The health outcome was the number of life years gained (LYs). Two costs were considered: the cost of administering TXA and the cost of additional days in hospital. Cost data were obtained from hospitals, World Health Organization (WHO) database and UK reference costs. Cost-effectiveness was measured in international dollars ($) per LY. Both deterministic and probabilistic sensitivity analyses were performed to test the robustness of the results to model assumptions.

**Findings:**

Administering TXA to bleeding trauma patients within three hours of injury saved an estimated 372, 315 and 755 LYs per 1,000 trauma patients in Tanzania, India and the UK respectively. The cost of giving TXA to 1,000 patients was $17,483 in Tanzania, $19,550 in India and $30,830 in the UK. The incremental cost of giving TXA versus not giving TXA was $18,025 in Tanzania, $20,670 in India and $48,002 in the UK. The estimated incremental cost per LY gained of administering TXA is $48, $66 and $64 in Tanzania, India and the UK respectively.

**Conclusion:**

Early administration of TXA to bleeding trauma patients is likely to be highly cost effective in low, middle and high income settings.

**Trial Registration:**

This paper uses data collected by the CRASH 2 trial: Controlled-Trials.com ISRCTN86750102, Clinicaltrials.gov NCT00375258 and South African Clinical Trial Register DOH-27-0607-1919.

## Introduction

The CRASH-2 trial showed that giving TXA to bleeding trauma patients results in a statistically significant and clinically important reduction in all-cause mortality (RR = 0.91, 95% CI 0.85 to 0.97), with no apparent increased risk of vascular occlusive events [1]. The trial inclusion criteria were clinical and did not depend on the results of laboratory tests. Patients were enrolled if they were judged by the doctor to have on-going significant haemorrhage, as evidenced by low blood pressure and a fast pulse, or if they were considered to be at risk of significant haemorrhage, for example, patients with compensated haemorrhage and stable vital signs, or those in whom bleeding might have stopped but who might start bleeding again following resuscitation. Further analyses have shown that the beneficial effects of TXA depend on the promptness with which TXA treatment is initiated. Early treatment, within about three hours of injury, appears to be more effective than later treatment. If administered within three hours TXA reduces the risk of death by 13% (RR = 0.87, 95% CI 0.81 to 0.95), while if TXA is administered after three hours it is not effective and can even been harmful [1].

On the basis of the CRASH-2 trial results, it has been estimated that the widespread use of TXA could save between 70,000 and 100,000 lives per year around the world. Because over 90% of trauma deaths are in low and middle income countries the potential for TXA to save lives is particularly high in these settings [2]. Nevertheless, TXA is unavailable in many low and middle income countries. Indeed, some hospitals in Africa that took part in the CRASH-2 trial, have reported that TXA is unavailable for routine clinical use. In high income countries, due to the low cost of the intervention, TXA is likely to be highly cost effective.

Although TXA is relatively inexpensive, evidence on the cost effectiveness of TXA in trauma is an important factor in the decision to include TXA on the WHO list of essential medicines. Economic evaluations of TXA in elective surgery show that TXA is a cost-saving intervention [3], [4]. A recent study of the cost effectiveness of TXA in four Sub-Saharan countries shows that in countries where there is either a shortage of blood, or where blood is not properly screened, TXA can reduce mortality. In countries where blood is available, TXA can reduce blood borne infections [4].

The objective of the present study is to assess the cost effectiveness of giving TXA to bleeding trauma patients. The cost of TXA and its effectiveness will vary between countries. In Low Income Countries (LICs), TXA is likely to cost less than in High Income Countries (HICs) because both administration cost and cost per day in a general ward are lower. However, in both LICs and Middle Income Countries (MICs) life expectancies are shorter and there will be a lower number of life years gained per patient. Using World Bank country classification criteria, the cost effectiveness of TXA was evaluated in three countries: Tanzania (LIC with Gross Domestic Product (GDP) per capita $509); India (MIC with GDP per capita $1,134); and the UK (HIC with GDP per capita $35,165) [5]. Tanzania was chosen in order to assess the cost-effectiveness of TXA when income per capita and life expectancy are low [6], [7]. India was selected because it is one of the MICs where TXA can avert the highest number of trauma deaths [2]. The UK was selected because within HICs it has an average GDP per capita and life expectancy.

## Materials and Methods

An economic evaluation was carried out to investigate the cost-effectiveness of administering TXA for the treatment of significant haemorrhage following trauma. Cost-effectiveness was measured by the incremental cost per life-year gained. The life-years gained are estimated from a simple Markov model where patients are either alive or dead, and by comparing the life-years experienced by a cohort who are given TXA with one that does not receive TXA. The model has an annual cycle and a lifetime horizon. Transitions between alive and dead in the first year are estimated from the CRASH-2 data, and in subsequent years from life tables. Life-years gained were discounted using an annual rate of 3.5%. The model was developed in Excel and STATA 11 software was used for the statistical analysis.

The cumulative risk of death during the first year in the placebo arm was estimated using CRASH 2 data. After the first year, patients were assumed to experience the same probability of death as the general population of a similar age, estimated using country specific life tables from WHO [8]. Assuming that TXA is administered within three hours (because after this time it is unlikely to be effective), the risk of death in the first year after trauma in the intervention group was calculated by multiplying the baseline cumulative hazard of the placebo group by the relative risk reduction of all cause mortality estimated in the CRASH-2 trial (RR = 0.87, 95% CI 0.81 to 0.95) [2]. Beyond twelve months the risk of death in the intervention arm was assumed to be equal to the one estimated for the placebo arm.

Since the CRASH-2 trial recorded data up to 28 days or death, a parametric survival function was fitted to extrapolate mortality experience over the twelve months following injury. Different parametric survival functions (Weibull, Gompertz, log-logistic, log-normal and generalised gamma) were compared using the Akaike Information Criterion (AIC), and Cox-Snell residuals were plotted as a confirmatory test [9]. The Gompertz model provided the best fit to the data. The Gompertz generic survival function is [9]:
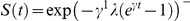
Where *t* is the time-frame over which the cumulative probability of survival is estimated, *γ* is an ancillary parameter that determines whether the hazard of death increases over time (if *γ* is positive) or decreases (if *γ* is negative). When covariates are considered in the analysis *λ* is given by the following equation [9]:
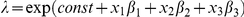
If data from the CRASH-2 trial are used the formula reported above becomes:
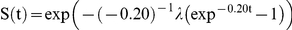
γ (−0.20, 95% CI −0.21 to −0.18) is negative meaning that after trauma the hazard rate decreases over time. Age, gender and GDP group were explored as covariates in the Gompertz model. A GDP per capita was assigned to each country in the trial according to the latest World Bank estimates and two binary variables, X_2_ and X_3_ were constructed to estimate whether the likelihood of death changes according to the GDP [6]. X_2_ took the value 1 for LICs and 0 otherwise. Similarly X_3_ took the value of 1 for MICs and 0 otherwise. As expected the baseline probability of death increases with age (age coefficient = 0.020, 95% CI 0.016 to 0.024) while gender was not found to be statistically significant (−0.06, 95% CI-0.22 to 0.11). Both GDP coefficients were found to be highly significant (β_2_ = −0.31; β_3_ = −0.61).

Thus, the function for *λ is*:
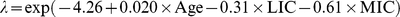
Since the number of life-years saved will depend in part on the age of the patient, the aggregate effect across all trauma victims was estimated by first running the model for different age groups, and then calculating the average result. Five age groups were identified each representing a similar proportion of the total trial participants: 16 to 20 years; 21 to 25 years; 26 to 34; 35 to 50; and more than 50 years. Patients in each group were assigned a starting age equal to the mean observed for that age group in the trial. The cumulative hazard rate, the probability of death in the first year after trauma, was estimated for each of these five mean ages. The overall number of life years saved in each country was then calculated as a weighted average of the results for the five age groups using the age distribution for trial participants in countries of the relevant income group.

**Figure 1:**
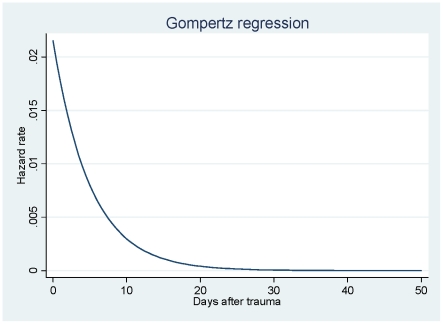
Hazard rate following trauma.

Figure 1 shows the extrapolated hazard rate function during the first fifty days after trauma modelled from the observed 28 day data for the placebo group in the trial. The modelled hazard rate decreases to almost zero in the first 28 days after hospital admission and then remains constant. This finding is consistent with previous studies suggesting that the majority of trauma-related deaths occur within a few weeks after injury. The figure also validates the model assumption that after one year the baseline risk of death is the same observed in the general population [10].

The incremental cost of TXA versus no TXA was calculated from a health service perspective [11]. Two cost items were considered in the present study: the cost of administering TXA to bleeding trauma patients and the incremental cost of non-intensive care hospital stay. Given that costs associated with TXA administration occur within one year post trauma, costs were not discounted. Costs have been converted from national currencies into international dollars ($) using Purchasing Power Parities (PPPs) [12].

In the main analysis, the cost of TXA ($5.70 per g) was taken from the British National Formulary and converted into international dollars [13], [14]. The same price was assumed for Tanzania and India in the absence of recent studies reporting the cost of TXA. The influence of different drug prices on the cost effectiveness of TXA was explored in one way and probabilistic sensitivity analyses. A dose of 2 g (1 g loading dose and 1 g maintenance dose) has been assumed as per the CRASH-2 trial protocol [2]. The CRASH-2 trial investigators estimate that the nursing time required to administer TXA ranges between 10–60 minutes. In Tanzania, a nurse's salary depends on both rank and on the location of the hospital. It can range between $800 per month, for a principal nursing officer, to $300 per month, for a newly started nurse (2008-9 prices) [15]. For the analysis, an average salary of $450 per month, which is the salary of a nursing officer, is assumed [15]. Using this estimate the average cost per hour was estimated to be $2.40 (range:$1.60–$4.20) [15], [16]. The average salary for a nurse in India varies by type of employer. Nurses working in public hospitals earn on average 30,000Rs ($2,044) per month while nurses employed in the private sector have a salary ranging between 7,000 and 10,500Rs per month ($480–$720) [17], [18], [19]. Assuming that a nurse works an average of 37.5 hours per week, the average cost per hour of a nurse in India was assumed to be $8.08 (range: $2.83–$14.06). In the UK, the average cost per hour of a nurse is $38 [13], [20]. The disposables used for TXA administration are two 10 ml syringes, two green needles, two bags of saline (100 ml and 500 ml for loading and maintenance doses respectively) and an IV administration set [21]. The cost of a syringe and needle was obtained from the study by Dziekan *et al*. [22] who estimated the average unit cost of syringes and needles in different regions of the world. The cost of saline and an IV administration set was obtained from the British National Formulary and converted into international dollars [14]. Storage and distribution costs per intervention are negligible. The overall cost of administering TXA per patient is estimated to be: $17.48; $19.55; and $30.83 in Tanzania, India and the UK respectively.

The improvement in survival following administration of TXA in the CRASH-2 trial was associated with a slight increase in the number of days spent in non-ICU hospital facilities (mean difference = 0.04, 95% CI 0.007 to 0.08, p = 0.0095). Since the incremental cost of non-ICU days associated with TXA will vary between countries, the mean length of stay of CRASH-2 patients in the placebo arm was calculated for low, middle and high income countries. As reported in Table 1, LICs patients tend to stay longer in the hospital.

**Table 1:** Resource use and Unit Costs.

Resource Use and Unit Cost	Tanzania	India	UK
**Resource use**			
TXA dosage (gram)	2	2	2
100 ml saline bag	1	1	1
500 ml saline bag	1	1	1
Minutes spent by nurse preparing & administering TXA	21	21	21
Average number of non-ICU days	10	7	8
**Unit Cost** ($)			
TXA per gram	5.70	5.70	5.70
Nurse per hour	2.37	8.08	38
Syringes and needles	0.18	0.19	0.23
IV administration set	4.35	4.35	4.35
Non-ICU hospital cost per day	13	28	429

Table 1 shows the unit cost of a non-ICU day. For Tanzania and India these were taken from WHO-CHOICE and uplifted to 2008-9 estimates, while for UK they were taken from Reference Costs [23], [24], [25], [26]. When more than one cost estimate was available per country, the average was used.

### Sensitivity analysis

One way sensitivity analysis was undertaken in order to assess the impact of uncertainty regarding the input parameters. The incremental cost per life year saved for TXA versus no TXA was estimated for different values of the RR of death with TXA (95% CI 0.81 to 0.95) [2] . One way sensitivity analysis was also conducted on the increase in non-ICU hospital stay following TXA administration (95% CI 0.007 to 0.08).

Due to the low cost of labour in both Tanzania and India, the drug cost constitutes almost 70% of the overall intervention cost. A cost-effectiveness analysis conducted by Casati *et al*. (1999) estimated that TXA would cost $2.57 per g, while a study conducted recently in India reported that TXA administration would cost $6.60 per g [27], [28]. In order to account for price variability between countries the cost effectiveness of TXA was estimated assuming a price ranging between $2.57, from the Casati *et al.* study, and $45.67 estimated by Eaton et al. [29]. Finally one-way sensitivity analysis was performed to estimate how the TXA cost effectiveness changes according to different estimates of the cost of a non-ICU day. The lowest estimates, for both Tanzania and India , were the cost per day in a primary health centre ($9.84 and $18.75 respectively) while the highest estimates ($18.69 and $35.41) were obtained from tertiary hospitals [23], [24]. In the UK, the minimum and the maximum non-ICU ward cost ($90–$784) were both obtained from UK reference costs [26].

Further sensitivity analyses were performed to evaluate how the results of the study change if different parametric distributions are adopted (Weibull, lognormal and log-logistic) when estimating survival beyond 28 days.

Monte Carlo simulations were used to explore further the robustness of the estimated cost-effectiveness. Uncertainty in the data was captured by fitting probability distributions to each parameter. The Beta distribution was selected for binomial data. All cost parameters, were assumed to follow a Gamma distribution while a Log normal distribution was chosen for relative risk parameters. Lastly, to reduce the uncertainty in the estimated survival parameters, a Cholesky decomposition of the covariance matrix was used to ensure that the parameters of Gompertz parametric model, γ and λ, were appropriately correlated on a log scale [30]. One thousand random samples were taken from all distributions and 1,000 estimates of the incremental cost, incremental life years saved and net monetary benefit were simulated. The probability that TXA was a cost-effective intervention was estimated by counting the proportion of simulations which produced positive net monetary benefits. Results were displayed with a cost effectiveness acceptability curve (CEAC) that shows the probability that the intervention is cost effective for different willingness-to-pay values for a life year saved.

## Results

### Base case analysis

Giving TXA increases costs because of the TXA administration cost and the longer non-ICU hospital stay (Table 2). The incremental cost of TXA per 1,000 patients is $18,025, $20,670 and $48,002 in Tanzania, India and the UK respectively (Table 2). The incremental cost of giving TXA is lower in Tanzania because both the personnel cost for administering the drug and the unit cost of non-ICU day are lower. As expected, the incremental cost of TXA is higher in the UK, where administering the drug to 1000 trauma victims would cost $30,830.

**Table 2:** Cost, Effectiveness and Cost-effectiveness of TXA in Tanzania, India and the UK.

Item	Tanzania	India	UK
**Non-ICU hospital stay ($)** *			
TXA	135,183	213,435	3,272,416
No TXA	134,641	212,315	3,255,244
**TXA administration cost ($)** *			
TXA	17,483	19,550	30,830
**Overall incremental cost ($)** *	18,025	20,670	48,002
**Life years gained discounted** *			
TXA	13,079	18,176	24,162
No TXA	12,707	17,861	23,407
**Incremental life year saved** *	372	315	755
**Incremental cost per life year saved ($)**	48	66	64

*per 1,000 patients.

Life years saved were estimated taking into account the age distribution of the trauma patients. The number of life years saved depends on both the baseline risk of death after trauma and on the life expectancy. TXA would save 372 LYs per 1,000 patients in Tanzania (which has lower life expectancy but a higher baseline risk of death). While in India TXA would save 315 LYs per 1,000 trauma patients. In the UK, where life expectancy is high, TXA can potentially save 755 LYs per 1000 patients. The incremental cost per life year saved is $48, $66 and $64 for Tanzania, India and the UK respectively (Table 2).

### One way sensitivity analysis

Results of one way sensitivity analysis are reported in Table 3. In all three countries, the price of TXA has a high impact on the cost effectiveness of the intervention. If the price of 2 g of TXA is $2.57 the cost-effectiveness of TXA would be $8 per LY (Tanzania), $12 per LY (India) and $26 per LY (UK). While, if TXA unit cost is $45.6 per patient, as suggested in the study conducted by Eaton et al. [29], TXA incremental cost per LY saved would be $124, $148, $86 in Tanzania, India and the UK respectively. Another important determinant of TXA cost effectiveness is the RR reduction associated with drug administration. If TXA was associated with a 19% reduction in the probability of death (lower bound of the 95% confidence interval) the incremental cost of TXA would be $33 in Tanzania, $45 in India and $43 in the UK. Neither the cost per day spent in a general ward, nor an increase in time spent in a general ward affect the cost effectiveness of TXA in Tanzania and India. In the UK, where the cost of a general ward is higher an increase in the mean length of non-ICU hospital stay would have a greater effect on the incremental cost-effectiveness ratio.

**Table 3:** One-way sensitivity analyses.

	Cost per life-year gained		
	Tanzania	India	UK
**TXA drug cost**			
$2.57	8	12	26
$45.6	124	148	86
**RR of death with TXA versus non TXA**			
0.81	33	45	43
0.95	126	170	168
**Additional non-ICU hospital stay for TXA patients**			
0.007 days	47	63	45
0.080 days	50	69	86
**Cost of non-ICU hospital stay (per day)**			
Low unit cost	47	64	46
High unit cost	49	67	82
**Parametric distribution used**			
Weibull	31	37	34
Lognormal	35	46	39
Log-logistic	25	42	32

The selection of the parametric distribution with which to extrapolate survival beyond study follow up can influence significantly the results of a cost effectiveness analysis [31]. The AICs and the Cox-Snell residual plots indicated that the Gompertz model was the best-fitting model (e.g. the AICs were 10087 (Weibull), 9997 (lognormal), 10064 (log-logistic) and 9459 (Gompertz)). Using different parametric distributions produced higher cumulative baseline hazards at one year and consequently lower cost per life year saved. For instance, the incremental cost per life year saved is $31, $37 and $34 in Tanzania, India and the UK respectively if a Weibull parametric function were to be adopted. The modelled gain in life years with the Gompertz model owes very little to the extrapolation beyond 28 days, the cost per life year saved increases from $48.4 to $48.6 (Tanzania), from $65.6 to $65.8 (India) and from $63.5 to $63.8 (UK).

### Probabilistic Sensitivity analysis

**Figure 2:**
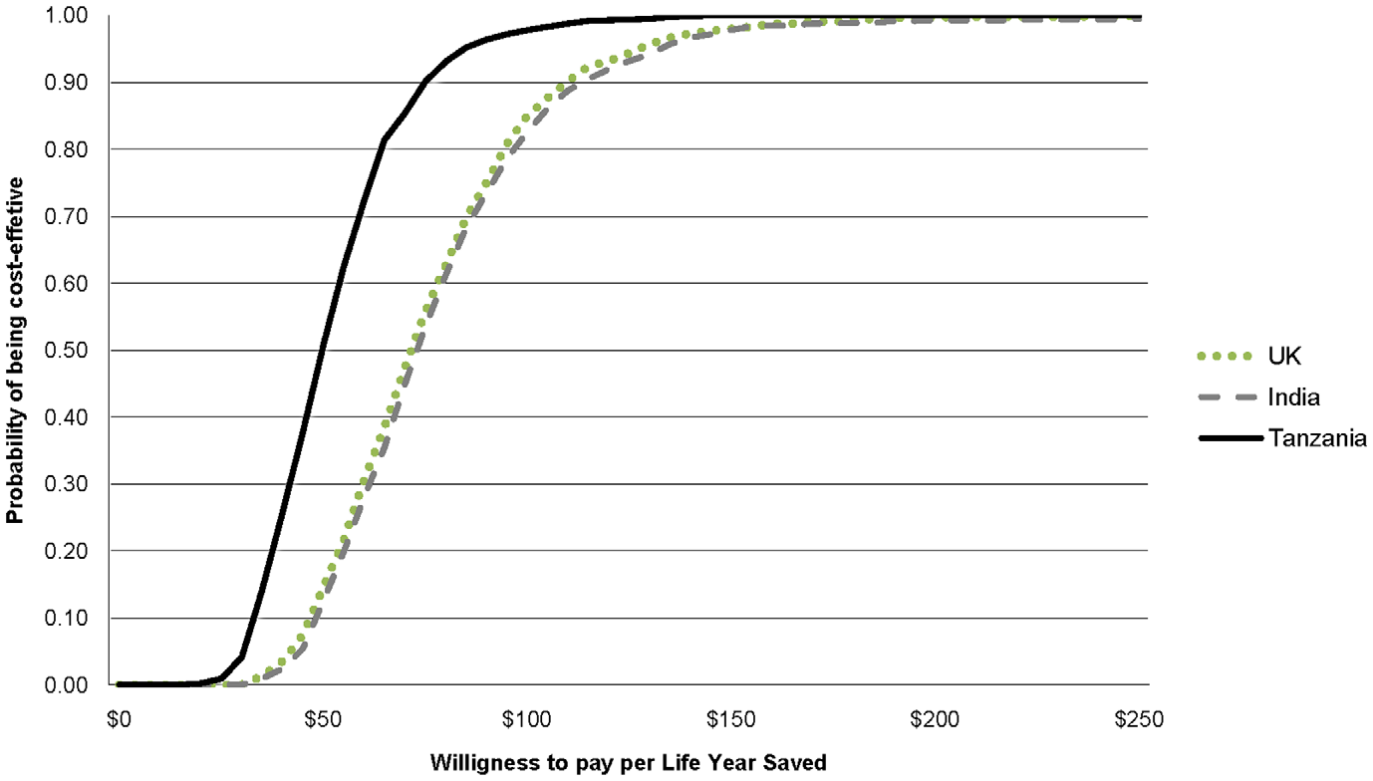
Cost-effectiveness acceptability curves showing the probability of TXA being cost effective in Tanzania, India and the UK.

The CEACs in Figure 2 show the probability that TXA will be cost effective for a range of willingness-to-pay values. Because of the cost of administering the drug and the incremental non-ICU stay, the routine administration of TXA is never a cost saving intervention. For any given willingness to pay between $25 and $250 there is a greater chance of TXA being cost-effective in Tanzania than in India or the UK. Overall, TXA is likely to be a cost effective intervention even if the willingness to pay for an additional life year saved is as low as $100.

## Discussion

This study evaluated for the first time the cost effectiveness of giving TXA to bleeding trauma patients in the UK, India and Tanzania, using primary data from the CRASH-2 trial which provides the best source of scientific evidence that TXA reduces the probability of death after trauma worldwide. This evaluation suggests that TXA is not only life saving, as shown by the CRASH-2 trial, but also that it is a highly cost effective intervention if administered routinely to bleeding trauma patients in high, middle and low income countries [2]. Early administration (within three hours) of TXA would cost $48, $66 and $64 per LY saved in Tanzania, India and the UK respectively. According to the WHO Commission on Macroeconomics, healthcare interventions costing less than GDP per capita per Disability-Adjusted Life Year (DALY) averted should be considered “very cost effective” [32]. DALYs are a measure of the years of life lost from disease and years lived with a disability. According to the World Bank classification, GDP per capita in low income countries ranges from $380 to $975, in lower middle income countries between $976 and $3855, upper middle income between $3,856 and $11,905 and high income countries GDP above $11,906 [33]. Thus, if the life years saved by TXA are spent in perfect health (one LY saved is equal to one DALY averted) TXA is a highly cost effective intervention in all the countries considered. The sensitivity analyses presented reinforce this conclusion.

However, a number of limitations should be considered when interpreting these results. It was necessary to model survival over 12 months using data for the first 28 days following the trauma and different statistical models will produce different estimates of benefit. However, different models were explored and the one selected as well as providing the best fit to the data also produced more realistic estimates of the impact on long term survival. In this evaluation those predicted to survive the first year following the trauma are assumed then to have the same life expectancy as members of the general population of similar age and gender.

A related limitation is that the measure of incremental cost-effectiveness is cost per life year gained rather than cost per quality-adjusted life year (QALY) (a year in perfect health is considered equal to one QALY) gained or DALY averted. Depending on the extent to which these patients do not enjoy perfect health, the cost per QALY gained or the cost per DALY averted will be higher than the cost per life year saved.

A further potential limitation is that the analysis does not allow for future health service savings. CRASH-2 showed that after 28 days the proportion of patients reporting no symptoms at discharge was significantly higher in the TXA group (14.7%) versus placebo (13.3%) (RR = 1.11, 95% CI 1.04 to 1.19, p = 0·0023) [2]. If TXA patients are more likely to survive without disability this study under-values the potential cost saving arising from the administration of TXA since healthier people will have lower future utilization of health care services.

Identifying cost effective interventions to decrease the number of injury related deaths is a major public health challenge. According to the Disease Control Priority Project in 2001 unintentional injuries alone account for 6% of all deaths worldwide [34]. The majority of unintentional injuries-related deaths, more than 90%, occur every year in low and middle income countries posing a further financial burden on these countries' economies and underfinanced health care systems [34].

This study suggests that TXA is a highly cost effective intervention in three very different settings. Given that TXA is effective and it is of relatively low cost it is not surprising that it is cost-effective in high income settings. However, it was important to demonstrate that it was likely to be cost-effective in countries with much more limited health care budgets, such as in Tanzania and India where due to the high numbers of trauma victims this simple intervention can avert thousands of deaths every year. Further research is needed to evaluate the effects of TXA on the quality of life of trauma patients.
